# Metabolomics for Clinical Biomarker Discovery and Therapeutic Target Identification

**DOI:** 10.3390/molecules29102198

**Published:** 2024-05-08

**Authors:** Chunsheng Lin, Qianqian Tian, Sifan Guo, Dandan Xie, Ying Cai, Zhibo Wang, Hang Chu, Shi Qiu, Songqi Tang, Aihua Zhang

**Affiliations:** 1 Graduate School and Second Affiliated Hospital, Heilongjiang University of Chinese Medicine, Harbin 150040, China; linchunsheng@hljucm.net (C.L.); guosifan612@163.com (S.G.); 13504555786@126.com (Y.C.); 13188465908@163.com (Z.W.); 2 Faculty of Social Sciences, The University of Hong Kong, Hong Kong 999077, China; tianqq@connect.hku.hk; 3 International Advanced Functional Omics Platform, Scientific Experiment Center, International Joint Research Center on Traditional Chinese and Modern Medicine, Hainan Engineering Research Center for Biological Sample Resources of Major Diseases (First Affiliated Hospital of Hainan Medical University), Key Laboratory of Tropical Cardiovascular Diseases Research of Hainan Province, Hainan Medical University, Xueyuan Road 3, Haikou 571199, China; dr.dan@hainmc.edu.cn (D.X.); qiushihnyx@163.com (S.Q.); tangsongqi@hainmc.edu.cn (S.T.); 4 Department of Biomedical Sciences, Beijing City University, Beijing 100193, China; bcbiyelunwen@163.com

**Keywords:** metabolomics, phenotype, biomarker, targets, mass spectrometry

## Abstract

As links between genotype and phenotype, small-molecule metabolites are attractive biomarkers for disease diagnosis, prognosis, classification, drug screening and treatment, insight into understanding disease pathology and identifying potential targets. Metabolomics technology is crucial for discovering targets of small-molecule metabolites involved in disease phenotype. Mass spectrometry-based metabolomics has implemented in applications in various fields including target discovery, explanation of disease mechanisms and compound screening. It is used to analyze the physiological or pathological states of the organism by investigating the changes in endogenous small-molecule metabolites and associated metabolism from complex metabolic pathways in biological samples. The present review provides a critical update of high-throughput functional metabolomics techniques and diverse applications, and recommends the use of mass spectrometry-based metabolomics for discovering small-molecule metabolite signatures that provide valuable insights into metabolic targets. We also recommend using mass spectrometry-based metabolomics as a powerful tool for identifying and understanding metabolic patterns, metabolic targets and for efficacy evaluation of herbal medicine.

## 1. Introduction

**Figure 1:**
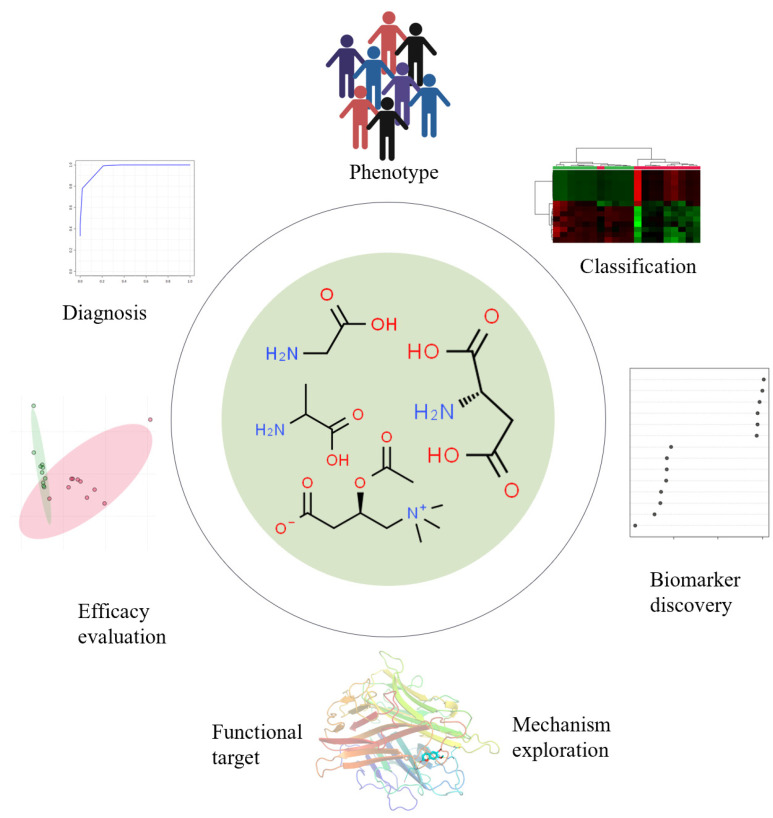
Potential roles of small-molecular metabolite biomarkers and diverse applications for discovery, diseases diagnosis, prognosis, monitoring treatments and efficacy evaluation. The images were obtained using the example data provided by the MetaboAnalyst 5.0 and the figures were created by BioRender (https://app.biorender.com/).

**Figure 2:**
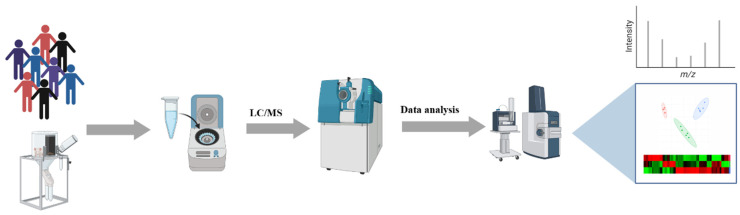
The flowchart of typical metabolomic analysis using LC/MS platform includes experimental design, sample collection, and data analysis stages. The images were obtained using the example data provided by the MetaboAnalyst 5.0, and the figures were created by BioRender (https://app.biorender.com/).

Metabolism is integrated with all biochemical reactions in bio-systems, and metabolomics provides metabolite information in biochemical processes [1,2]. Abnormal metabolites can be used as potential biomarkers for biomarker discovery, evaluating diagnosis, and monitoring treatment; the potential role of small molecule metabolites is shown in Figure 1. Metabolomics involves the systematic quantification and identification of small-molecule metabolites and is a global analysis of metabolite profiles of complex biological systems. High-throughput metabolomics can rapidly develop and identify small metabolites and molecular mechanisms to improve disease diagnosis and treatment and reveal disease etiology and treatment targets. Recently, it has been a pivotal tool for understanding the relationship between phenotypic conditions and biochemical processes and has emerged as an effective approach for the holistic analysis of small metabolites in biological systems [3,4,5]. Its most common application, coupled with mass spectrometry, statistical, bioinformatics, and algorithm tools, is used for screening of disease-specific diagnosis biomarkers, specifically elucidating the composition and biological effects of drugs [6,7,8]. The flowchart of typical metabolomics analysis includes experimental design, sample collection, and data analysis stages, as shown in Figure 2.

Bioactive compounds have gained great attention in industrial and pharmacological fields. Herbal medicine (HM) could produce beneficial effects and treatments for various diseases. However, its complexity currently hinders research progress, including unclear mechanisms of action, unclear active ingredients, unidentified therapeutic targets, and advances in drug metabolism. This complexity makes it extremely challenging to scientifically reveal its efficacy and mechanism of action. Recently, the mass spectrometry-based metabolomics method has been widely used for analysis of herbal compounds [9,10,11]. It is an important tool to reveal the efficacy and mechanism of herbal medicines from the perspective of small-molecule metabolism and promote the discovery of herbal drug discovery [12,13].

The main challenges in disease diagnosis, prognosis, and treatment are the lag in biochemical indicator detection and uncertainty in therapeutic efficacy. A metabolome is a collection of small-molecule metabolites in organs or cells, used as biomarkers for diagnosing or predicting diseases. The metabolome consists of all small-molecule metabolites and often reflects phenotypic variations closest to the phenotype of living organisms, providing important clues to understand biomedical mechanisms contributing to various symptoms, indicating that it may be a powerful approach to reveal the action mechanism of HM [14]. The identification and validation of sensitive and robust biomarkers and targets are also required. Nowadays, MS-based metabolomics technology is the key method to identify various changes at the small-molecule level [15]. MS-based metabolomics can be as used as a preliminary discovery, testing and clinical diagnostics prototype for clinical practice in the near future. The advantages of MS-based metabolomics are discussed with a focus on its developing role in target discovery and demystifying herbal treatment. This work also summarizes an overview of the high-throughput MS-based metabolomics techniques used in research of the key metabolites and metabolic pathways, efficacy evaluation, active ingredient discovery, multi-target and mechanism exploration, and specifically highlights current and future applications.

## 2. Advanced Mass Spectrometry

**Figure 3:**
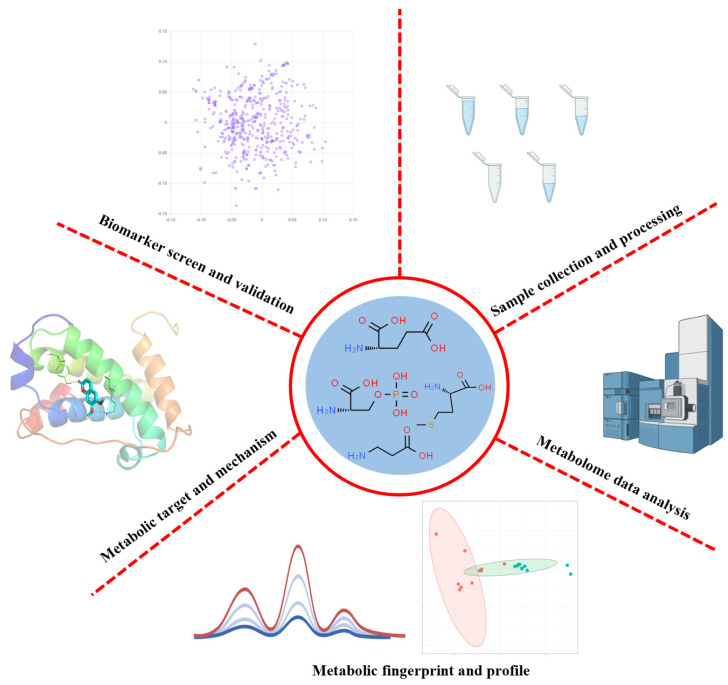
Overview of analytical workflow of small molecule metabolite-based metabolomics. The images were obtained using the example data provided by the MetaboAnalyst 5.0, and the figures were created by BioRender (https://app.biorender.com/).

Metabolomics, the comprehensive research of a metabolome, actively grasps the analytics of pathways and networks of small metabolites and covers a range of small metabolite molecules with molecular weights less than 1500 Da, including sugars, lipids, amino acids, nucleic acids, organic acids, fatty acids, etc. [16,17]. Due to the diverse and complex structures of small-molecule metabolites, detecting metabolism remains the most challenging task in metabolomics research. Mass spectrometry has been used for rapid detection of small-molecule metabolites for understanding the metabolic network changes from whole organisms to single cells. Due to the metabolite complexity, there is no single analytical platform that can be used to detect all metabolites in biological samples, and a combination of different analytical techniques could obtain a wide range of metabolomics. Spectral techniques include mass spectrometry (MS), combined chromatography such as liquid chromatography (LC), capillary electrophoresis (CE), gas chromatography (GC), supercritical fluid chromatography (SFC), and nuclear magnetic resonance (NMR) spectroscopy, etc. Each technology has unique advantages in accuracy, sensitivity, reproducibility, and resolution, etc. Due to the sensitivity of LC-MS to thermally unstable, non-volatile substances, its application scope has exceeded that of GC-MS and NMR. In recent years, LC/MS has become the most popular analytical platform to improve identification of metabolites and analyze the versatility of metabolites [18,19]. Technological advances in mass spectrometry-based metabolomic analysis platforms pave the way for disease biomarker discovery, diagnosis, prognosis, efficacy evaluation, active ingredients, targets and mechanism exploration (Figure 3). Mass spectrometry has been increasingly implemented in various fields in drug monitoring, toxicology, herbal medicine and microbiology and is becoming an indispensable tool to gain insights into multiple aspects of living systems [20,21]. Its advancement has largely facilitated small-molecule metabolite exploration, which provided a general picture of metabolic changes related to disease alteration. Comprehensive metabolome analysis could contribute to active-ingredient discovery, multi-target mechanism studies and efficacy evaluation. Spatial omics based on mass spectrometry imaging with microscopy will provide new ideas for searching for active ingredients, therapeutic targets, and revealing the effective mechanisms of natural products [22,23,24]. At present, the high-throughput mass-spectrometry imaging technology is an effective analytical method that can simultaneously visualize and spatially detect, quantify, and image small metabolite molecules to understand complex communication networks. Metabolomic data can be automatically processed using bioinformatics tools. A wide range of online resources are available, including metabolite identification (e.g., Metlin), biostatistics tools (e.g., MetaboAnalyst), and public data repositories (e.g., HMDB, KEGG).

## 3. Mass Spectrometry-Based Metabolomics for Metabolic Phenotype Signatures

Metabolite signatures are closely related to disease phenotype. A metabolome is composed of downstream products of genome, transcriptome and proteome, and the most proximal correlate to the body phenotype. High-throughput mass spectrometry-based metabolomics could characterize the metabolic phenotypes with respect to human health, disease, and even drug monitoring. The metabolic phenotype of chronic obstructive pulmonary disease evolves during pulmonary rehabilitation, and methanol is correlated with a strict relationship with clinical parameters [25]. By analyzing gut microbiota and serum metabolites, the metabolic phenotype of chronic heart failure is established for screening of elderly patients [26]. Inflammatory arthropathies and osteoarthritis are causes of significant morbidity in the world. Gluconic acid, glycolytic-acid and tricarboxylic acid-related substrates were elevated in osteoarthritis patients, while cardiolipins and glycosphingolipids were elevated in rheumatoid arthritis patients [27]. In rheumatoid arthritis patients, metabolite profiling of metabolites provided insights into circulating pro-/anti-inflammatory metabolic signatures and enabled the discovery of biomarkers for risk factors, clinical subgroups, quantitative disease activity and response predictors of treatment (Hur et al., 2021). Untargeted metabolic profiling discovered the differential regulation of the tryptophan, urea cycle/amino group, aspartate/asparagine, tyrosine, and lysine involved in systemic inflammation in coronary artery disease patients [28].

The ceramides in acute myocardial infarction patients were associated with cardiometabolic risk [29]. Plasma samples were analyzed by flow-injection liquid chromatography-mass spectrometry and showed that branched-chain α-keto acids and glutamate/glutamine were metabolic biomarker signatures of insulin resistance in childhood obesity [30]. The increased levels of lauric acid were associated with the severity of COVID-19 patient infection [31]. Tryptophan, the kynurenine/tryptophan ratio, serine and threonine might be early biomarkers of peripheral artery disease with a high risk [32].

Amino acid metabolic profiles of moyamoya disease were developed; L-glutamic acid, o-phosphoserine, L-methionine and β-alanine showed high specificity and sensitivity [33]. A non-invasive metabolomics method was used for the diagnosis of kidney disease in type II diabetes mellitus and found that triglylcarnitine, dodecanoylcarnitine and isovalerylcarnitine serve as the potential biomarkers [34] (Abdelsattar et al., 2021). Targeted metabolomics showed that the glucose, 1-methlynicotinamide and glycine involved in nucleotide synthesis and energy metabolism were significantly altered in prostate cancer (PCa) patients [35]. Serum metabolomic profiling of ankylosing spondylitis patients revealed the perturbations of amino acid metabolism in serum. Specifically, the differential metabolites lysine, serine, proline and alanine were correlated with disease activity [36]. Glutamate was significantly increased, and decenoylcarnitine was decreased in patients with relapsing–remitting multiple sclerosis [37]. Decanoylcarnitine, tetradecadienylcarnitine, and pimelylcarnitine can obviously predict a lower risk of Alzheimer’s dementia phenotypes [38].

## 4. Potential Biomarkers Discovery by Mass Spectrometry

**Figure 4:**
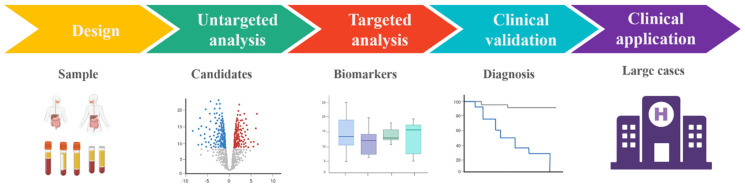
General workflow of metabolic analysis as a driver for biomarker discovery and validation of small-molecule metabolites. Figure created by BioRender (https://app.biorender.com/).

Metabolites has translational value in disease prediction and can offer insights into metabolic pathways and new mechanisms associated with disease. Figure 4 shows the general workflow of metabolic analysis as a driver for biomarker discovery and validation of small-molecule metabolites. 2,5-di-tert-butylhydroquinone and 13-HOTrE(r) in cerebrospinal fluid and arachidonoyl PAF, 3-tert-butyladipic acid, and 1-methyluric acid in serum were regarded as biomarkers of vascular cognitive impairment [39]. Non-targeted metabolomics was performed and showed that lower levels of norvaline and L-aspartic acid as well as 1,5-anhydroglucitol were related to the progression of macroalbuminuric diabetic kidney disease [40]. Small-molecule metabolites in plasma were investigated by liquid chromatography-mass spectrometry. 4-Pyridoxate, d-glutamic acid and xanthine could serve as non-invasive biomarkers for pulmonary tuberculosis [41]. Total metabolites were screened as potential biomarkers and related metabolic pathways including sphingolipid metabolism, retinol metabolism, lysine degradation, glycerophospholipid metabolism, etc. [42]. Serum metabolites of type 2 diabetes were profiled using non-targeted metabolomics with mass spectrometry and revealed that a total of five metabolites (piperidine, cyclohexylamine, stearoyl ethanolamide, N-acetylneuraminic acid, 1,2-distearoyl-glycero-3-phosphocholine) are obviously increased in type 2 diabetes complications [43].

Diabetes is a chronic disease influencing millions of people in the world and imposes huge costs on society. Using the targeted technique, the acylcarnitines and amino acids that reflected the metabolic disturbances were found as potential biomarkers for preventing, diagnosing, and treating diabetes [44]. Combinations of targeted and pseudotargeted metabolomics analyzing serum metabolites of pediatric lymphoma patients, carnitine, creatine, leucine, and taurine, etc., provide diagnosis and targets for pediatric lymphoma. Distinct organic and amino acid profiles were processed to identify small metabolites of epithelial ovarian cancer, and glutamine, methionine, asparagine, glycolic acid and glutamic acid were identified as marker metabolites [45]. A targeted metabolomics approach was used to reveal serum biomarkers, such as sulfoxy methionine, glutamate, aspartate, glutamine, asparagine and methionine, as potentially useful biomarkers for noninvasive and rapid diagnosis of pulmonary tuberculosis [46]. A total of 14 potential biomarkers were identified in coronary heart disease (CHD), and amino acids, energy metabolism and fatty acid metabolism were correlated with the severity of coronary stenosis in CHD patients [47]. Significantly decreased concentrations of deoxycholic acid, chenodeoxycholic acid and cholic acid were identified as potential biomarkers and associated with end-stage renal disease patients [48]. Serum metabolites including 2-deoxygalactopyranose, 2-dodecylbenzenesulfonic acid, and L-pipecolic acid, were significantly perturbed in refractory tumor-induced osteomalacia [49]. Serum metabolomic profiles based on liquid chromatography-mass spectrometry were used for early screening of differential metabolites in infants with sepsis, and prolylhydroxyproline was found to have high diagnostic value as a potential biomarker [50].

## 5. For Disease Diagnosis and Classification

Cancer has various effects on metabolism at systemic levels [51]. Small metabolites hold a great promise for diagnosis, monitoring and therapy of disease in clinical and translational research [52]. For early breast cancer diagnosis, the metabolites [caprylic acid; ethyl (*R*)-3-hydroxyhexanoate; hypoxanthine] could form an effective diagnostic model [53]. Panel biomarkers including aspartic acid, glutamate, and proline have been identified as potential diagnostic biomarkers of oral squamous cell carcinoma [54]. During the discovery and validation stages, significant alterations in aspartate, alanine, and glutamate metabolic pathways highlighted potential metabolic pathways for early diagnosis of cervical cancer [55].

Several small-molecule biomarkers that were associated with kidney complications have been reported for prediction and diagnosis of diabetic patients. Specifically, bile acids were associated with lipid measurements and glycemic control in the diabetes clinic [56] (Ahonen et al., 2019). There were six panels of biomarkers for distinguishing the diabetic patients, which might be useful for the diagnosis of prediabetes and diabetes complications [57]. Comprehensive metabolomics was conducted to discover 11 differential metabolites as early biomarkers for diabetic kidney disease [58].

A panel consisting of small-molecule metabolites in urine combined with clinical indicators could serve as useful biomarkers for diagnosis of polycystic ovary syndrome phenotypes [59]. Candidate biomarkers of intraductal papillary mucinous neoplasms for early diagnosis were identified and included carboxylic acids, amino acids, conjugated bile acids, trimethylamine-oxide and purine oxidation products [60]. Metabolism analysis of plasma samples was performed to identify and discover peripheral biomarkers in patients with major depressive disorders. The increased diethanolamine, L-aspartic acid, and alanine, and the decreased cystine, O-acetyl-L-carnitine and fumarate were promising biomarkers for major depressive disorder diagnosis [61]. To discover AD biomarkers in the hair by mass spectrometry metabolomics, a study identified a metabolic panel of nine biomarker candidates in the early stage, indicating high potential for early detection of AD dementia [62]. Ultra-performance LC-MS metabolomics was used to determine metabolic changes in steroid-sensitive nephrotic syndrome and obtained a total of 194 differential metabolites and 5 related metabolic pathways [63].

3′-Deoxy-3′,4′-didehydro-cytidine in serum is an accurate biomarker for acute viral infection diagnostics and could generate an AUC (area under curve) of 0.954 [64]. Nonalcoholic steatohepatitis was related to the changed levels of cholesterol metabolites involved in detoxification and inflammation induction [65]. A novel biomarkers pattern including arginine/ornithine, arginine, hydroxylbutyrylcarnitine and vaccenylcarnitine was established and achieved an AUC of 0.89 for the differential diagnosis of acute ischemic stroke [66]. It is difficult to differentiate between asymptomatic hypercholinaemia of pregnancy and intrahepatic cholestasis of pregnancy. T-ω-MCA and glycocholic acid were regarded as diagnostic biomarkers, and Gtri-S-2 and TLCA-S were combination biomarkers for differential diagnosis (AUC was 0.990) of hypercholanemia and intrahepatic cholestasis of pregnancy [67].

Tissue-based spatial metabolomics was analyzed the metabolic signatures to classify gastric cancer patients [68]. Serum biomarkers of early-stage bladder cancer detected by liquid chromatography-high resolution mass spectrometry included L-octanoylcarnitine, threoninyl-alanine, PGF2a ethanolamide and showed good discrimination performance [69]. Mass spectrometry analysis of seven metabolites exhibited higher diagnostic and discriminating capacity [70]. Membrane phospholipid signatures are promising biomarkers, revealing the highest discrimination between hepatocellular carcinoma tumors and liver tissue [71].

Due to the urgency of the COVID-19 pandemic, metabolomics applications in infectious diseases is an evolving area of science. The AUC predictive ability of molecular features as potential biomarkers was validated in plasma and urine samples, reaching 0.904 and 0.988, respectively [72]. Urinary metabolic markers have promising potential in distinguishing asymptomatic patients from healthy controls and predicting the incidence of high-risk sequalae in COVID-19 patients [73]. Many differential metabolites in COVID-19 patients at different stages of progression were identified as biomarkers, including propylparaben, triethanolamine, 20-hydroxyeicosatetraenoic acid, chavicol, disialosyl galactosyl globoside, alpha-methylstyrene, etc. [74]. Serum metabolite pathways of type 2 diabetic retinopathy, proliferative diabetic retinopathy and non-proliferative diabetic retinopathy revealed the pathways of linoleic acid metabolism, alanine metabolism, and arginine biosynthesis metabolism which were dysregulated in diabetic retinopathy patients; glutamate, glutamine, aspartate and N-acetyl-l-glutamate were increased; icosapentaenoic and docosahexaenoic were decreased and could distinguish between non-proliferative diabetic retinopathy and proliferative diabetic retinopathy patients. 13(S)-hydroperoxylinoleicacid and phosphatidylcholine were identified as metabolite biomarkers in advanced stages of diabetic retinopathy [75]. Asn-Cys-Pro-Pro and Asn-Met-Cys-Ser were relevant physiopathologic biomarkers and related to progression of DKD proteinuria [76].

## 6. Disease Prognosis Analysis via Mass Spectrometry-Based Metabolomics

Significantly higher levels of phosphatidyl ethanolamine, phosphatidylcholine (PC), phosphatidylserine, phosphatidylmethanol, and diacylglyceryl trimethylhomoserine and phosphatidylethanol that related to lipid metabolism could assist clinicians in the prognosis of pancreatic cancer patients [77]. Untargeted metabolomics revealed that lipids including PC (30:2) and PC (30:1) as biomarkers of prognosis play a key role in discriminating early hepatocellular carcinoma and compensated cirrhosis [78]. Lactate was identified as an essential molecule and reinforced Treg cells in the tumor microenvironment via lactylation [79]. The circulating level of gut microbe-generated metabolite trimethylamine N-oxide (TMAO) was associated with the risks of various cardiovascular diseases and can be used as a mediator biomarker for monitoring the severity and prognosis of atherosclerotic vascular disease [80]. Mass spectrometry was used to evaluate the abnormal metabolites related to prognosis of multiple myeloma patients and to identify the significant metabolic disorders [81]. A predictive model associated with COVID-19 severity and prognosis was constructed by a set of immunological and metabolomic indicators, such as troponin I, neutrophil/lymphocyte ratio, malic acid, etc. [82]. Liquid chromatography-mass spectrometry metabolomics as a promising approach analyzed sepsis-induced cardiac dysfunction and found that kynurenic acid and gluconolactone could be used as metabolite markers for diagnosis and prognosis for septic patients with cardiac dysfunction [83].

## 7. Mass Spectrometry-Based Metabolomics for Revealing Pathological Mechanism

Biomarker discoveries could facilitate the targeting of pathophysiological signatures [84]. High-throughput MS metabolomics can identify small molecule biomarkers and explain the complex pathomechanism of disease. Oleic acid could promote the proliferation and migration of colon carcinoma cells and play a key role in CRC development and treatment [85]. The kynurenine pathway metabolites in Alzheimer’s disease (AD) patients are considered to be involved in the neuropathogenesis of AD and as therapeutic targets for patients with AD [86]. Compared to cognitively unimpaired individuals, small-molecule TMAO in cerebrospinal fluid is higher in AD dementia individuals and is associated with phosphorylated tau and neuronal degeneration. It provides an understanding of the gut-brain axis [87]. From serum metabolic changes, the potential differential metabolites were identified in hyperuricemia patients, which provided associations of amino acids, organic acids, fatty acids, lipids profiles of hyperuricemia individuals, and shed light on its etiology and pathogenesis process [88]. LC-MS-based metabolomic profiling screened out and identified 240 candidate biomarkers in chronic pelvic inflammatory disease and enriched in glyoxylate metabolism, steroid hormone biosynthesis, the glucagon pathway, etc. [89]. Metabolic disorder changes in malic acid, petroselinic acid, and methionine sulfoxide contributed to the progression of sepsis [90]. Long-chain fatty acids serve as an important energy source and as signaling molecules and could play key roles in cellular energy metabolism [91]. Serum signatures at the acute phase suggest arginine and tryptophan metabolism as key pathways in COVID-19 patients, providing evidence of the underlying mechanisms of COVID-19 disease pathogenesis.

## 8. Mass Spectrometry-Based Metabolomics for Monitoring Treatments

Monitoring treatment of disease progression is particularly important for the patient. Non-targeted metabolomics was used to identify metabolic changes in human non-alcoholic fatty liver disease after exercise intervention and revealed that alterations in amino acid metabolism may play a key role in improving fasting blood glucose concentrations in plasma [92]. Pharmacologic correlation analysis revealed the therapeutic mechanisms of ellagic acid that play antipyretic anti-inflammatory effects via inhibiting five metabolic pathways and the IKB-α/NF-κB signaling pathway [93]. Short-chain fatty acids have beneficial effects in intestinal health and as signaling molecules that affect the host metabolism. The intestinal microbiota affecting the metabolic phenotype of the host was mediated via short-chain fatty acids regulating the function of active organs, mainly the small-molecule metabolites propionate acetate and butyrate, which were derived from microbial fermentation of indigestible carbohydrates [94]. TMAO derived from gut microbial metabolites has been implicated in cardiovascular disease pathogenesis and could facilitate development of therapeutic interventions [95]. Metabolomics profiling using gas chromatography mass spectrometry can predict the efficacy of chemotherapy and immunotherapy in patients with non-small cell lung cancer and shows that key metabolic pathways in response to chemotherapy and immunotherapy include glutamate metabolism, the glucose-alanine cycle, the urea cycle, etc. [96].

The perturbations in amino acid and sphingolipid metabolism may provide valuable information on therapeutic targets for the early treatment of anorexia nervosa [97]. Mass spectrometry-based phenotypic analysis identifies small-molecule metabolite panels correlating with radiation therapy, showing high correlations (AUCs ≥ 95%) and predicting radiation therapy [98]. A cell-active small-molecule tool has identified glucose-6-phosphate dehydrogenase as a target for modulating immune responses [99]. The small-molecule 1,5-anhydro-D-glucitol that binds to the SARS-CoV-2 spike protein is beneficial in the fight against COVID-19 in people with diabetes [100]. Nontargeted and targeted metabolomics using liquid chromatography with mass spectrometry explored the amino acid metabolome and revealed the potential utility of key amino acids involved in multiple myeloma disorders, providing more evidence for understanding therapeutic targets [101]. Studies have shown that bile acids and polyunsaturated fatty acids are associated with the chronicity and severity, respectively, of drug-induced liver injury (DILI). Ratios of omega-6/omega-3 PUFAs and primary/secondary bile acids were increased in DILI patients. A combined model of aspartic acid and adrenic acid was established and has excellent performance for predicting DLIL chronicity [102]. Four metabolic pathways of citrate cycle, glycerophospholipid, pyruvate metabolism and sphingolipid were metabolic aberrations that impact the pathogenesis of multiple sclerosis and provide clues for developing potential treatment strategies [103].

## 9. Modulating Metabolism Analysis Using Mass Spectrometry

Investigating the metabolic features of diseases has identified many potential treatment options. Mass spectrometry-based metabolomics profiling analysis has highlighted metabolic pathways that may serve as potential therapeutic targets for disease treatment. Precision editing of metabolism and composition in gut microbiota could decrease the tumor risk and prevent the development of colitis-associated colorectal cancer [104]. Active regulation of the microbiota metabolism composition by tungstate treatment could ameliorate dysbiosis in inflammatory diseases [105]. Targeting tryptophan catabolism has found therapeutic potential as a key metabolic regulator of cancer progression [106]. A total of 1137 small-molecule metabolites were screened in the human brain, and tryptophan metabolism was identified as an endogenous regulator targeting the pathogenesis of AD [107]. Primary fatty-acid amides and glutamate in plasma were related to hippocampal volume and memory for AD [108]. Higher succinate and citrate in plasma are associated with neurocognitive functions, supporting the importance of altered metabolism in the pathogenesis of neurocognitive impairment [109]. Targeting gut-derived metabolites is an effective approach to improving the symptoms associated with autism spectrum disorder [110]. Gut bacteria-derived indole-3-propionic acid metabolite can facilitate functional recovery and regeneration of sensory axons by an immune-mediated mechanism [111]. The increased concentrations of microbially derived tryptophan and propionate can cause mitigation of gastrointestinal syndromes, long-term radioprotection, and reduction in proinflammatory responses [112]. Different metabolites among the uterine serous carcinoma and endometrial cancer were discovered, and most belonged to purine nucleotides and amino acids, including 2-oxo-4-methylthiobutanoic acid synthesized via the acireductone dioxygenase 1 enzyme, which are potential therapeutic targets in poor-prognosis serous carcinoma patients [113].

## 10. Therapeutic Target Discovery via Mass Spectrometry-Based Metabolomics

Metabolism is integrated with all biochemical changes, and most diseases can result in the pathological metabolic changes that play a key role in disease progression. Through analyzing the metabolic changes, mass spectrometry-based metabolomics can reveal the potential functional target or promising enzymatic targets for therapeutic intervention. Metabolomics serve drug discovery methods, integrating the metabolome into a functional omics to develop new drugs and screen new metabolic targets. In particular, the application of LC/MS targeted metabolomics has led to the discovery of new metabolic targets and novel biomarkers in biological systems for understanding disease mechanisms, effective substances, and therapeutic targets. It is used as a drug target discovery strategy and has been successfully applied to the development of HM natural products and therapeutic drugs. Functional target discovery of bioactive lead compounds from natural products is key to understanding effect mechanisms, due to their biological activities and various structures. Mass spectrometry-based metabolomics is an effective strategy to establish innovative methods for discovering the natural product targets.

The metabolic alterations may help the discovery of functional target and biomarkers. Untargeted metabolomics analysis showed that glycerophospholipid metabolism was associated with the tumorigenesis of esophageal squamous cell carcinoma (ESCC) [114]. Glycerophospholipid metabolism was considered to be a therapeutic target of ESCC progression. Mass spectrometry-based metabolomics could offer novel prognostic biomarkers and elucidate the progression mechanisms of diabetic kidney disease [115]. Via unlabeled and labeled metabolomic analysis, several metabolites were identified in diabetic kidney disease progression. The metabolomic profiles and potential disease-specific biomarkers of cystic renal disease patients were established, respectively [116]. Glutathione, TCA cycle, aminoacyl-tRNA biosynthesis and amino acid metabolism have the most impact. The early treatment could target aspartate, alanine, and glutamate pathways. Untargeted metabolomics was used for analyzing disease biomarkers of allergic asthma, determining the serum metabolomic profiles and therapeutic monitoring [117]. Metabolomic analysis revealed that 15(S)- and 12(S)-HETEs can be involved in the treatment of allergic asthma and can serve as new therapeutic targets for monitoring allergic asthma. GC-MS-based metabolomics has speculated that metabolic alteration is vital for ameliorating polycystic ovary syndrome [118]. Metabolic phenotypic analysis based on GC-MS showed that targeted amino acid pathways have a better therapeutic effect on pain with symptomatic knee osteoarthritis [119]. Liquid chromatography–mass spectrometry metabolomics profiling revealed the metabolite changes associated with aging and found that dysfunction of branched-chain amino acid metabolism and mitochondrial membrane-related lysophospholipids were determined to be promising therapeutic targets for aging [120].

Untargeted serum metabolomics were performed to screen candidate biomarkers between lung cancer and healthy control. It showed 15 metabolite markers were dysregulated, and it demonstrated that cholesterol, 4-hydroxybutyric acid, myo-inositol, oleic acid and 2-hydroxybutyric acid have excellent diagnosis ability through multiple algorithms [121]. It revealed that combinations of these small metabolites could be promising biomarkers for early lung cancer. Metabolic profiling was also capable of identifying candidate biomarkers that could enable detection of breast cancer (BC) [122]. By analyzing the untargeted and targeted datasets, metabolic pathway analysis showed eight potential metabolic pathways that were significantly altered in early BC. The inositol phosphate metabolism and aminoacyl-tRNA biosynthesis were most impacted and provided key information regarding underlying mechanisms in early BC. Untargeted metabolomics revealed eighteen altered metabolites in rheumatoid arthritis (RA) [123]. Using random forest algorithm and receiver operator characteristic curve analysis, a three-metabolite panel including citric acid, L-glutamine, and L-cysteine was constructed. Additionally, an integrated analysis revealed dysregulation of the taurine biosynthetic pathway as the pathophysiological mechanisms and providing alternative therapeutic targets for RA.

## 11. Efficacy Evaluation of Herbal Medicine and Natural Products

**Figure 5:**
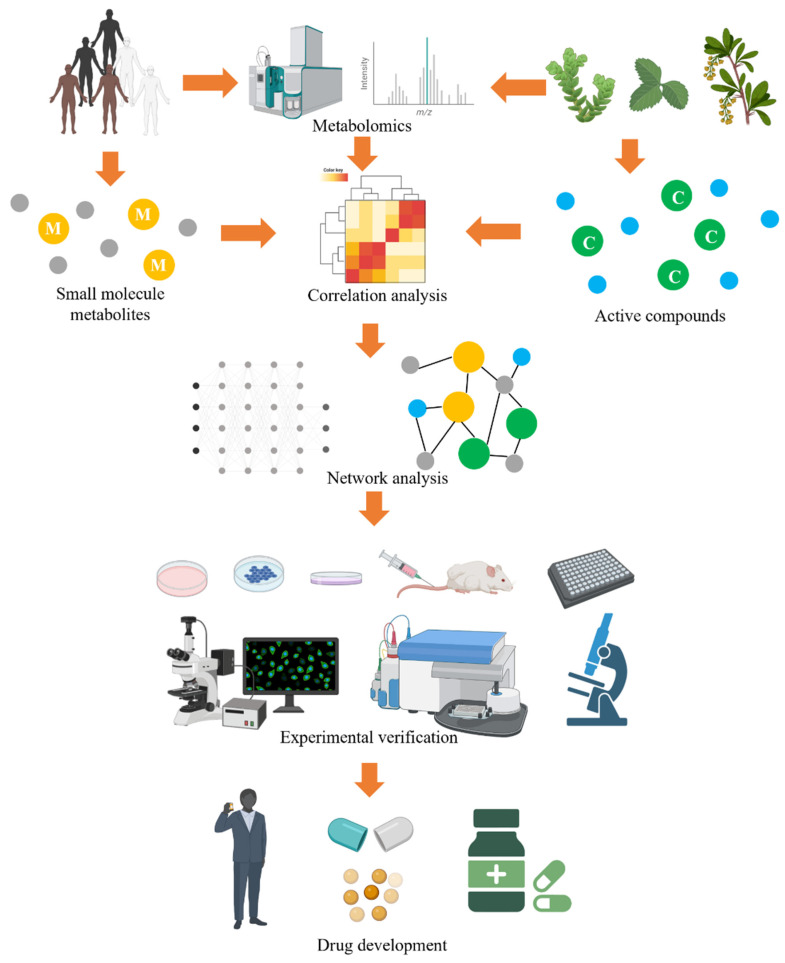
Active ingredient discovery and development of medicinal plants and the schematic illustration of herbal medicine and natural products investigation by integration technologies. The images were obtained using the example data provided by the MetaboAnalyst 5.0, and the figures were created by BioRender (https://app.biorender.com/).

Metabolic alterations often lead to disease states and, thereby contributing to various biochemical processes that contribute to disease symptoms. Mass spectrometry-based metabolomics is of great significance for the discovery of metabolic targets and metabolic regulators for elucidating the metabolic mechanisms. It has emerged as an invaluable tool to understand the HM effect at the small-molecule metabolite level and has been applied in herbal medicine research related to drug responses, biomarker discovery and target discovery associated with pharmacological effects and therapeutic mechanisms (Figure 5).

Integrated UPLC/MS metabolomics revealed the reno-protective effect of Radix Preparata and Rehmanniae Corni Fructus on chronic kidney disease (CKD) rats [124]. In total, 15 different metabolites play a key role in CKD progress and are involved in the related bile acid metabolism, amino acid metabolism, and glycerophospholipid metabolism. The therapeutic effect of Mori Fructus polysaccharide (MFP) against acute alcoholic liver injury can be achieved by regulating key metabolic pathways, including α-linolenic acid, linoleic acid, and glycerolphospholipid metabolism [125]. The results indicate that MFP can exert hepatoprotective effects associated with improved lipid metabolism. Metabolomics methods based on UPLC/MS revealed the effects of a *Saposhnikovia divaricata* decoction (SD) [126]. A total of 13 and 18 potential biomarkers for SD treatment were identified in urine and plasma, respectively. SD could alleviate symptoms of RA and effectively regulate the disturbed metabolic glycerophospholipid catabolism, tryptophan, fatty acid, and primary bile acid biosynthesis. Metabolomics combined with UPLC/MS explored the therapeutic effects of a Gandou decoction (GDD) against hepatolenticular degeneration [127]. After treatment, it was suggested that GDD can achieve a therapeutic effect by participating in metabolic disturbances with the disordered metabolites such as lysoPE (20:2/0:0), sphinganine, and taurochenodesoxycholic acid.

High-throughput mass spectrometry-based metabolomics was applied to explore therapeutic effects of a Korean herbal decoction Gyejibongnyeong-Hwan (GBH) in blood stasis (BS)-induced shoulder pain [128]. The results showed that the ratio of kynurenine to tryptophan was one of the indicators related to pain generation. Metabolomic analysis found that Phillygenin (PHI), an important component of *Forsythiae fructus*, could alleviate liver fibrosis by promoting the production of SCFAs and restoring the disturbance of BA metabolism [129]. Fecal metabolomics could uncover the laxative effects of a traditional herb, Rhubarb [130]. After rhubarb treatment, the chenodeoxycholic acid, α-linolenic acid and cholic acid were notably increased and showed a close relationship between fecal metabolites and intestinal flora. Untargeted metabolomics has investigated the anti-diabetic beneficial effects of red ginseng extract [131]. A total of 50 biomarkers indicated an adjustment trend after intervention with red ginseng extract, and the regulated pathways included D-glutamine metabolism, taurine and hypotaurine metabolism, and tryptophan metabolism.

A urine metabolic profile showed that sebacic acid, glutaric acid, 3-methylhistidine, allantoin, L-isoleucine, and caprylic acid were identified as potential biomarkers of depression and are involved in the aminoacyl-tRNA biosynthesis, tyrosine metabolism, isoleucine biosynthesis, fatty acid biosynthesis, etc. [132]. A combination of sebacic acid, allantoin, L-isoleucine was screened out as efficacy markers associated with the pharmacodynamic effect of *Millettia speciosa Champ*. Targeted metabolomics was to evaluate anti-aging efficacy and screen the effective extracts of Erzhi Wan (EZW) [133]. Intermediates of energy synthesis and TCA cycle pathways were used to identify marker metabolites for revealing the therapeutic effect of EZW. Urinary metabolomics identified the biomarkers associated with hyperlipidemia and revealed the underlying antihyperlipidemic effect of a Chinese patent medicine, a Daming capsule [134]. Most of the biomarkers were regulated to normal levels by the Daming capsule, and its lipid-lowering effect is related to the regulation of amino acid metabolism, the tricarboxylic acid cycle, and the pentose phosphate pathway. The effect of a *Schisandra chinensis–Radix ginseng* herb pair on Alzheimer’s disease was explored [135]. Sixteen endogenous metabolites, including tryptophan metabolism and purine metabolism, were identified as potential biomarkers. For Alzheimer’s disease treatment, it mainly regulates the energy metabolism, gut microbiota and neurotransmitters, and reduces inflammation. A total of 25 differential metabolites were related to the effects of *Rhizoma Coptidis* extracts on acute kidney injury [136]. It has protective effects on the metabolism regulation and nuclear factor-erythroid 2-related factor-2 pathways.

**Figure 6:**
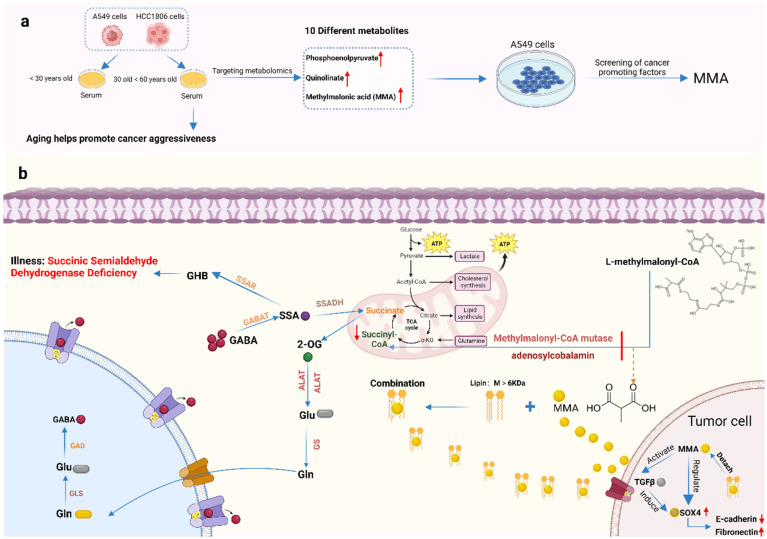
Metabolomics for biomarker discovery and therapeutic target identification. Pathogenesis of disease based on regulation of metabolites in the tricarboxylic acid cycle. (**a**) Targeted cancer promotion experiment on the metabolites showed that methylmalonic acid (MMA) was the main cancer-promoting factor in aging serum. (**b**) Cancer-promoting mechanism of MMA found that MMA is produced when the conversion of L-methylmalonyl-CoA to Succinyl-CoA is abnormal. MMA is difficult to transmembrane itself and binds with lipoproteins (>6 kDa) to transmembrane into cancer cells. The figure was created by BioRender (https://app.biorender.com/).

The ultra-high performance liquid chromatography coupled with tandem mass spectrometry metabolomics was used to explore the therapeutic mechanisms of schisandrol A against pulmonary fibrosis and revealed that TGF-β1-VIM-carnosine and TGF-β1-ID3-creatine pathway were key pathways of Sch A modulating the metabolic disorders [137]. Serum metabolomics combined with UPLC/MS analyzed the effective substances and action mechanisms of Ziziphi Spinosae Semen extract for insomnia treatment [138]. A total of 21 endogenous biomarkers were screened out, involving tryptophan, tyrosine biosynthesis, and nicotinate metabolic pathways, and 34 chemical constituents, such as magnoflorine, N-nornuciferine, oleic acid, coclaurine, ceanothic acid, palmitic acid, and betulinic acid were identified as potential effective compounds for insomnia treatment. Active ingredient discovery and the development of medicinal plants from the small-molecule metabolite perspective are shown in Figure 6. An integrated herbal metabolomic analysis was used for rapid discovery of quality markers in *Gardenia Fructus*, and then to screen out the pharmacological components [139]. A total of 5 active components were identified and highly correlated with liver protection, and 15 active components were identified with anti-inflammatory properties. Geniposide, gardenoside and genipin-1-β-D-gentiobioside were found as quality markers of Gardenia Fructus. Mass spectrometry and targeted metabolomics were used to reveal key components and find more medicinal values of *Chaenomeles speciosa* (Sweet) Nakai (*C. speciosa*) fruits [140]. A total of 974 metabolites were identified and 548 differential metabolites were screened. The plant metabolomics analysis as an effective approach showed nine ingredients could be considered as quality markers of *Periplocae Cortex* [141] Metabolomics combined with mass spectrometry imaging methods were used to study metabolic markers to explore the differences between processed and raw Fuzi [142]. Quantitative metabolomic analysis showed flavonoid treatment could decrease arachidonic acid (AA) levels [143]. Mangiferin, isomangiferin and neomangiferin as anti-inflammatory components could inhibit AA metabolism. An integrated UPLC/MS metabolomics was used to discover bioactive ingredients of *Eucommiae folium* (Ef) in treating kidney injury [144]. The results indicated that Ef could regulate the vascular endothelial growth factor, hypoxia-inducible factor 1, and glycerophospholipid pathways.

## 12. Multi-Target and Effect Mechanism Exploration of Herbal Medicine

High-throughput mass spectrometry-based metabolomics has been used to investigate the underlying mechanisms of Qishen granules on myocardial ischemic chronic heart failure and revealed that Qishen granules might alleviate amino acid metabolism disorders by regulating endogenous metabolites [145]. UPLC/MS metabolomics analysis revealed that the Hu’po Anshen decoction regulated the abnormalities of glucose-alanine and malate-aspartate shuttle to promote bone formation [146]. A urine metabolomics showed that the Baitouweng decoction can effectively treat dampness–heat diarrhea by regulating the differential metabolites involved in tryptophan metabolism, glutathione metabolism, and methionine metabolism [147]. Metabolomics showed eleven metabolites as biomarkers of the antipyretic mechanism of ellagic acid, mainly involving glycerophospholipid and sphingolipid metabolism, etc. [148]. Mass spectrometry-based metabolomics was employed to discover biomarkers and mechanisms after agarwood intervention. A total of 18 potential biomarkers were closely related to insomnia and involved in tryptophan metabolism, steroid biosynthesis, and arginine metabolism pathways [149]. Results showed that sesquiterpenes and 2-(2-phenylethyl) chromones could exert high docking abilities with target proteins. Integrative metabolomics based on mass spectrometry explored the possible anti-arthritis mechanism of Yaobitong capsules on adjuvant-induced rheumatoid arthritis rats [150]. A total of 32 potential biomarkers were related to amino acid and nucleotide, lipid, and glucose metabolism. The down-regulated 3-hydroxy-hexadecanoic acid, 2-oxoarginine, and the up-regulated l-glutamic acid have high predictive ability for rheumatoid arthritis.

Multi-target and mechanism explorations of herbal medicine and natural products can be studied through innovative integration technologies. An integrating metabolomics was established to explain the blood-supplementation mechanism of *Angelica sinensis* (AS), which was related to linoleic acid, l-cystathionine, aspartic acid and l-alanine metabolic pathways [151]. Metabolomics analyses have identified EGCG and myricitrin as α-glucosidase inhibitors from dried leaves of *Syzygium polyanthum* leaves [152]. Rabdosia Serra (RS) is the dried aerial parts of *Rabdosia serra* (Maxim.) *Hara*. A metabolomics platform that explored the underlying pharmacological mechanisms of RS in cholestatic rats [153], and revealed that 26 biomarkers and the biosynthesis of unsaturated fatty acids, arachidonic acid, and tryptophan metabolism pathways were regulated by RS treatment. The inflammation processes and bile acid secretion process are the key biochemical reactions in RS treatment, which could protect the liver by promoting bile acid excretion and by regulating the imbalance of key metabolic pathways. Metabolomics has been used to explore the potential anti-fatigue mechanism of *Radix Salviae miltiorrhizae* [154]. Metabolic pathways altered by potential biomarkers include glutathione metabolism, the TCA cycle, glucose metabolism, glyoxylate and dicarboxylic acid metabolism. It has anti-fatigue effects by regulating the oxidant–antioxidant balance and energy metabolism. A metabolomics evaluation was used to reveal the complicated anti-psoriatic mechanism of LiangXueJieDu from a holistic perspective [155]. It can improve the metabolic pathways of steroid hormone biosynthesis and glycerophospholipid metabolism.

Non-targeted metabolomics has been used to clarify the pharmacological mechanism of 5-Hydroxy-4-methoxycanthin-6-one (PQ-A) for colitis treatment [156]. The study found 49 differential metabolites involved in the perturbation of bile acid metabolism, sphingolipid metabolism, alpha-linolenic acid metabolism, etc. A total of 36 marker metabolites were markedly regulated by PQ-A via the disturbed metabolic pathways. Plasma metabolomics analysis has explored underlying mechanisms of a well-known traditional herb, hawthorn, and found that it acts by modulating amino acid metabolism, lipid metabolism and energy metabolism [157]. It was applied to uncover the therapeutic mechanism of *Gardenia jasminoides* fruits on type 2 diabetes [158]. After treatment, the pathologic symptoms were significantly ameliorated, and some metabolite biomarkers were regulated and involved in amino acid metabolism, bile acid biosynthesis, etc. A metabolomics approach was performed to investigate ameliorative effects of *Bidens bipinnata* L. on hyperlipidemic rats [159] and found that six endogenous metabolites were involved in linoleic acid metabolism. Metabolomics has been used for biomarker discovery and therapeutic target identification, such as the regulation of methylmalonic acid (MMA) metabolites, a main cancer-promoting factor in aging serum (Figure 6).

## 13. Limitations and Challenges of Mass Spectrometry-Based Metabolomics

As we have shown, small molecular metabolites have much potential, from laboratory to clinical applications. However, translating these molecules from the laboratory to the clinic remains a follow-up challenge. So, there are still some challenges and limitations of mass spectrometry-based metabolomics for small-molecular metabolites that need to be addressed in the field of small-molecule metabolites. Metabolic datasets influenced by sampling techniques and analytical methods can lead to significant variability. However, future challenges will include the standardization of metabolic datasets, experimental protocols, and data analysis software to better explore potential molecular mechanisms. Proper sample processing and pre-analysis procedures are crucial, because several analytes are prone to in vitro distortion during sample collection [160]. Therefore, it is necessary to establish standardization of laboratory procedures for sample extraction, sample processing, and data mining. In addition, this standardization process using mass spectrometry-based metabolomics should be reflected at each stage. Due to the high complexity of mass spectrometry metabolomics, a major limitation is the inaccurate identification of biomarkers of metabolites related to phenotypic variations, which can solve the problem of detecting specific molecules through special statistical software, multivariate recognition patterns, or bioinformatics tools, thereby analyzing accurate mass and fragment-mass spectrometry data. Another limitation is the lack of validation of metabolic biomarkers of disease and lack of specificity and sensitivity [161]. We should mine out accurate and sensitive metabolites and conduct more experiments to verify these findings. In addition, there is still a need to clinically validate metabolite biomarkers in a large population. One of the biggest challenges is mainly in the field of data integration and requires further consideration. In order to achieve this goal, with the help of artificial intelligence and computational algorithm technology, the integration of multiomics can improve the coverage of low-abundance metabolites, helping to accurately identify small metabolites and related biological disturbances in the body. Due to the high complexity of a metabolome, there is no unified analysis approach that can analyze all metabolites with metabolic phenotypes. Fortunately, integrating multiple omics techniques can provide insights into the metabolic changes in diseases.

## 14. Conclusions

**Figure 7:**
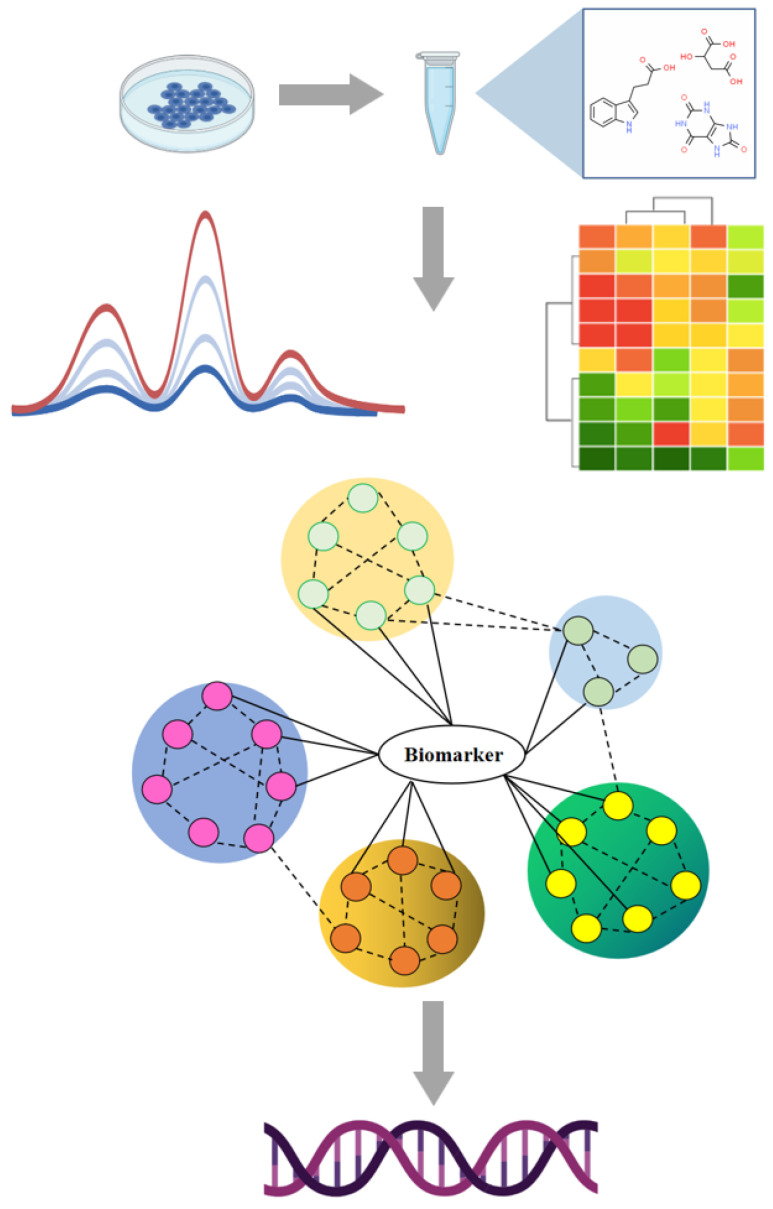
Functional interpretation and action mechanism investigation of small-molecule metabolites. The figures were created by BioRender (https://app.biorender.com/).

Mass spectrometry-based metabolomics for small-molecular metabolites could provide abundant evidence for early disease diagnosis and prognosis as well as monitoring responses. Future studies should validate metabolite markers discovered by mass spectrometry-based metabolomics to improve the understanding of abnormal metabolic mechanisms. Mass spectrometry-based functional metabolomics could precisely capture the interactions between disease phenotypes and metabolite biomarkers, open new insights to reveal the close correlation between small metabolites and functional genes with metabolic phenotype, and then to unravel regulatory processes and metabolic networks (Figure 7). Targeted and untargeted metabolomics strategies are effective approaches in biomedical science, especially applied in disease-related biomarkers and drug development. However, small-molecule metabolites require multi-cycle and large-sample verification, from clinical to experimental and from experimental to clinical. This overview emphasizes the current applications of mass spectrometry-based metabolomics in target discovery and demystifying herbal treatments. The current article analyses the value of mass spectrometry-based metabolomics as a driver for biomarker screening and highlights the vast complexity of target discovery to analyze HM, particularly to shed light on the investigation of bioactive compounds, targets and therapeutic potential. It provides successful examples from efficacy evaluation, active ingredient discovery to multi-target and mechanism exploration of herbal medicine, and also demonstrates an effective road-map for the demystifying of HM for the benefit of patients and healthcare in the future.

## List of Abbreviations

Herbal medicine (HM); mass spectrometry (MS); liquid chromatography (LC); capillary electrophoresis (CE); gas chromatography (GC); supercritical fluid chromatography (SFC); nuclear magnetic resonance (NMR); liquid chromatography combined with mass spectrometry (LC/MS); prostate cancer (PCa); coronary heart disease (CHD); phosphatidylcholine (PC); trimethylamine N-oxide (TMAO); Alzheimer’s disease (AD); drug-induced liver injury (DILI); breast cancer (BC); rheumatoid arthritis (RA); chronic kidney disease (CKD); Mori Fructus polysaccharide (MFP); Saposhnikovia divaricata decoction (SD); Gandou decoction (GDD); Gyejibongnyeong-Hwan (GBH); blood stasis (BS); Phillygenin (PHI); Erzhi Wan (EZW); arachidonic acid (AA); Eucommiae folium (Ef); methylmalonic acid (MMA; Angelica sinensis (AS); Rabdosia Serra (RS).
